# Microbiome-Related Amino-Acid Profiles of Liver and Muscle Tissues in Arbor Acres Broiler Chickens

**DOI:** 10.3390/biology15201806

**Published:** 2026-10-10

**Authors:** Dmitry Deryabin, Lyudmila Vlasenko, Ilshat Karimov, Dianna Kosyan, Shamil Rakhmatullin, Galimzhan Duskaev

**Affiliations:** 1 Laboratory of Breeding and Genetic Research in Animal Husbandry, Federal Research Centre of Biological Systems and Agrotechnologies, 460000 Orenburg, Russia; dgderyabin@yandex.ru (D.D.); ifkarimov@yandex.ru (I.K.); kosyan.diana@mail.ru (D.K.); 2 Department of Epidemiology and Infectious Diseases, Orenburg State Medical University of the Ministry of Health of Russia, 460014 Orenburg, Russia; 3 Department of Feeding for Farm Animals and Feed Technology, Federal Research Centre of Biological Systems and Agrotechnologies, 460000 Orenburg, Russia; shahm2005@rambler.ru (S.R.); gduskaev@mail.ru (G.D.)

**Keywords:** broiler chickens, amino-acid profile, gut microbiome

## Abstract

The study, designed as a group-level integrative data analysis, included homogeneous datasets obtained from 24 Arbor Acres chicken groups. Quantitative amino-acid analysis in chicken tissues was carried out using the capillary electrophoresis method. Gut microbial community analysis was performed using 16S rRNA gene-sequencing technology on the Illumina MiSeq platform. Amino-acid analysis of liver samples revealed 10 Arg-high and 14 Arg-low chicken groups, while for thigh samples, the ratio of Arg-high and Arg-low groups was 18/6, respectively. The gut microbiomes of chickens with an Arg-high amino-acid profile showed increased Chao1 and Shannon diversity index values, defined by a significant abundance of several members of the phylum *Bacillota* (formerly *Firmicutes*), including the genera *Christensenella*, *Eisenbergiella*, *Dysosmobacter*, *Anaerotignum*, *Oscillibacter*, unclassified *Oscillospiraceae*, and *Lachnospiraceae*. In contrast, the gut microbial communities of chickens with low Arg content showed decreased Chao1 and an increased Shannon dominance index, which was explained by the significant abundance of the genus *Alistipes* belonging to the phylum *Bacteroidota* (formerly *Bacteroidetes*). Prediction of amino-acid biosynthetic pathways in chicken-gut microbiomes revealed a general trend toward increased relative abundance of most KEGG orthologs in Arg-high groups, as well as a number of positive links with Arg and Val/Leu + Ile content in liver and thigh samples.

## Simple Summary

This study aimed to analyze the relationships between amino-acid profiles in broiler chicken tissues and chicken-gut microbiome composition, developing the concept of the “gut microbiome–liver–muscle” axis. Comparative amino-acid analysis revealed two distinct profiles in liver and thigh samples that differed significantly in arginine content, while no such differences were found in breast samples. Significant associations were found between arginine-high and arginine-low profiles in chicken tissues and the gut microbial communities described in the categories of biodiversity indices, the abundance of specific bacterial taxa, and reconstructed amino-acid metabolism pathways. This insight confirms the possibility of modulating amino-acid content in broiler chickens by targeting their gut microbiome composition.

## 1. Introduction

Chicken meat and edible chicken byproducts are a major source of dietary animal protein worldwide [1]. Favored for their advanced production technologies, relatively low price, and adaptability to a variety of regional cuisines, they represent a cornerstone of global food supply [2]. According to an FAO report, poultry production reached approximately 137 million tons in 2020, with chicken accounting for more than 90% of this total output [3]. Currently, global chicken production continues to grow rapidly to meet the increasing demand for affordable and high-quality animal protein [4].

The nutritional value of chicken proteins lies not so much in their moderate energy content, but in the variety of amino acids (AAs) that are the building blocks for muscle growth, tissue repair, and overall human health [5]. Notably, chicken meat and byproducts contain nine essential amino acids (EAAs), which cannot be synthesized de novo and are vital for daily metabolic functions, with over 40% of the total amino-acid content in chicken proteins being EAAs [6]. In addition, among the EAAs, three branched-chain amino acids (BCAAs) are usually distinguished, which are crucial for muscle protein synthesis and energy metabolism, and which serve as signaling molecules that regulate blood sugar levels and immune system function [7]. In turn, among the nonessential amino acids (NEAAs), it is customary to distinguish flavor-related amino acids (FRAAs) responsible for the key taste characteristics of food, glycine, alanine, and serine, which provide sweet notes, as well as glutamate and aspartate, which add the savory (umami) taste [8].

Several endogenous and exogenous factors can significantly impact the AA content in chicken tissues, and they are fundamentally dictated by a complex interplay of genetic selection, age, sex, dietary formulation, and environmental conditions [9].

The profound influence of genetic selection is determined by changes in GC content affecting gene expression patterns [10], mutational bias as a driving force for codon usage in the *Gallus gallus* genome [11], and a number of other genetic mechanisms that alter protein deposition and the AA profile in fast-growing commercial hybrids compared to unselected local chicken lines [12]. Differences in AA content between chickens of different ages [13] are presumably related to the expression of intestinal amino-acid transporters, which is largely dependent on the developmental stage of the bird, altering AA absorption and assimilation efficiency [14]. Furthermore, AA distribution varies drastically across different chicken tissues, particularly those interacting via liver–muscle metabolic crosstalk [15]. According to this concept, the liver is the primary site of AA and amino-acid precursor uptake from the chicken gut for further de novo synthesis and catabolism, which are then distributed to the thigh and breast muscles, which also exhibit notably different AA profiles [16].

Among external factors, the most studied is nutrition, which focuses on diet supplementation with free AAs, the combination and balance of which directly determine AA content in the chicken tissues [17]. Another important environmental factor is ambient temperature fluctuations, including heat stress, which triggers the liver to catabolize circulating amino acids for energy rather than send them to muscle tissue for protein deposition [18].

A novel factor influencing AA content and protein quality in chickens is the gut microbiome, which acts as an interface between the external environment and host physiology [19]. In current knowledge, gut microbiota play an important role in final protein digestion, de novo AA synthesis, and their efficient absorption by the host, which promotes improved feed conversion ratios and increased growth rates [20]. Several studies have documented potential benefits of the *Lachnospiraceae* family (including the genus *Lachnoclostridium*), which was positively associated with higher EAAs (Met, Tyr, Leu) and FRAA (Glu) content, meat fiber diameter, and chicken body weight [21,22]. In turn, in Yang et al.’s study [23], abundance of the genus *Turicibacter* was negatively correlated with muscle Asp and Tyr content, whereas the genus *Parabacteroides* was positively correlated with Met, and numerous other links were found between different bacterial taxa and specific AAs. Growing evidence of the gut microbiome–liver–muscle axis [24] provides a possibility to modulate AA metabolism and improve poultry meat quality using probiotics [25] or dietary supplements [22] targeting the gut microbiota community.

The goal of this study was to identify the chicken-gut microbiome characteristics, including biodiversity indices, key bacterial taxa, and metabolic pathways related to AA profiles in the liver, thigh, and breast muscles. To exclude the role of genetic factors, all experiments were carried out on a single line of the fast-growing Arbor Acres strain, while the role of external factors was minimized using a basic diet standardized in AA content, as well as universal poultry farm conditions. In turn, the variability of the examined microbiomes was a consequence of the use of dietary supplements (probiotics or plant-derived molecules) targeted specifically at the microbial community in the chicken gut.

## 2. Materials and Methods

### 2.1. Ethics Statement

The study protocols were approved by the Animal Ethics Committee of the Federal Research Center of Biological Systems and Agrotechnologies of the Russian Academy of Sciences, following Article 2(d) of the European Convention for the Protection of Vertebrate Animals used for Experimental and other Scientific Purposes (18 March 1986) and the principles of good laboratory practice.

### 2.2. Experimental Design and Animal Management

The study was designed as a group-level integrative data analysis format and included multiple homogeneous quantitative data sets obtained from multiple chicken groups, previously described in [26,27,28,29,30,31,32]. The study was aimed at identifying associations between the gut microbiome and the amino-acid profiles of broiler chicken tissues that were not evident in each experiment performed for the same variables using similar measurement protocols.

Animal trials were carried out at the vivarium in the Federal Research Center of Biological Systems and Agrotechnologies, Orenburg, Russia, between 2019 and 2024. A total of 24 groups were represented by the Arbor Acres strain purchased from a local supplier (Aviagen LLC, Tula, Russia). After the post-hatch period, 7-day-old male broiler chickens were randomly divided into 3 control and 21 experimental groups of 15–30 poults each, marked with plastic foot tags and arranged in specialized cages with an area of 4050 cm^2^ (90 × 45 × 45 cm, 3 tiers) with a mesh floor. The temperature was maintained at 26–28 °C for 2–4 weeks and then gradually reduced to 20–22 °C for 5–7 weeks. Relative humidity was maintained at 65–70% throughout the entire time. The photoperiod program (23 h continuous light 30 L: 7 D, 20 h continuous light 10 L: 8–20 D, and 18 h continuous light 8 L: 21–45 D) complied with the European Council Directive (2007/43/EU), which regulates the protection of chickens kept for meat production.

According to Arbor Acres Broiler Nutrition Specification (2022) (https://aviagen.com/assets/Tech_Center/BB_Foreign_Language_Docs/RUS_TechDocs/AA_BroilerNutritionSpecifications2022_RU.pdf, accessed on 30 August 2026), the chickens received a two-phase basal diet (BD): the grower diet from days 8–28 and the finishing diet from days 29–42. The granulated compound feed was represented by pellets of 2.5–3.0 mm. The control groups received BD only, while the experimental groups were fed BD supplemented with antibiotic chlortetracycline [26,27], *Bacillus*-containing probiotic additives [28], or chemically synthesized analogues of the following plant-derived molecules: gamma-lactone, coumarin derivative, vanillin/vanylic acid, resorcinol derivative, or quercetin [26,27,28,29,30,31,32]. The working hypothesis underlying the current study was that the variability in the examined microbiomes was a consequence of the use of dietary supplements that specifically targeted the microbial community in the chicken’s gut, while the supplements themselves had no direct effect on amino-acid metabolism in the host, or their effect was not obvious.

### 2.3. Sample Collection

At the end of the experiments (on day 42), 3 chickens from each replicate were randomly selected, humanely euthanized, and immediately aseptically dissected. Their liver (typically the caudal part of the right lobe), thigh (central part of the *iliotibialis* muscle), and breast (central part of the *pectoralis major* muscle) samples collected from each chicken were placed in separate plastic tubes and preserved at −20 °C for amino-acid analysis. The gut (cecal) contents were massaged into sterile cryogenic tubes, snap-frozen in dry ice, and stored at −80 °C in an 803CV freezer (Thermo Fisher Scientific, Waltham, MA, USA). The downstream individual AA measurements were averaged to generate one group-level value for liver, thigh, and breast samples; the cecal samples were pooled.

### 2.4. Analysis of the Amino Acids Content in Chicken Liver and Meat Samples

Quantitative amino acids analysis in broiler chicken tissues was performed using a validated capillary electrophoresis method M-04-94-2021, designed by Lumex Instruments Ltd. (Mission, BC, Canada).

The samples preparation was as follows: (i) liver, thigh and breast samples were dried to constant weight at 60 °C; (ii) 100 mg of each sample was placed into a glass vial (ALWSCI Technologies Co., Ltd., Shaoxing, China) and hydrolyzed in 10 mL of 6 N HCl at 110 °C for 14–16 h to break down proteins into free amino acids; (iii) after filtration through slow-flow 2–3 µm pore size cellulose discs, 0.05 mL of the mixtures was transferred to glass tubes for drying in a stream of warm air; (iv) free amino acids were transformed to phenylthiocarbamyl (PTC) derivatives using the Edman’s reagent under alkaline conditions (pH 8.5–9.0) at room temperature for 35 min; (v) the mixture was dried again, dissolved in 0.5 mL of distilled water and centrifuged at 5000 rpm for 5 min.

The resulting highly stable, UV-absorbing PTC derivatives were measured using a “Capel-105M” electrophoresis system equipped with a special capillary cassette for amino-acid analysis (Lumex, Saint Petersburg, Russia). The ionic forms of PTC derivatives were separated in a quartz capillary (Leff/Ltot 65/75 cm; ID 50 μm) at +25 kV in phosphate buffer (pH 7.4) with 0.37% β-cyclodextrin. The PTC derivatives were determined by measuring their absorbance at 254 nm.

The method enables fast quantitative determination of the following amino acids: histidine (His), serine (Ser), glycine (Gly), phenylalanine (Phe), tyrosine (Tyr), alanine (Ala), valine (Val), lysine (Lys), methionine (Met), threonine (Thr), proline (Pro), and arginine (Arg). Because leucine and isoleucine are not clearly separated by electrophoresis, they are measured in total (Leu + Ile). The results of quantitative amino acids measurement are presented as *w*/*w* values.

### 2.5. DNA Extraction, PCR Amplification, and 16S rRNA Gene Sequencing

Total genomic DNA was extracted from gut samples using the commercial Fast DNA SPIN Kit for Feces (MP Biomedicals Inc., Irvine, CA, USA) or QIAamp Fast DNA Stool Mini Kit (Qiagen GmbH, Hilden, Germany) according to the manufacturer’s protocols. The extracted DNA quality was evaluated by 1% agarose gel electrophoresis, and DNA concentration was assessed using the dsDNA high-sensitivity assay kit for a Qubit 4 fluorometer (Life Technologies, Carlsbad, CA, USA).

The hypervariable V3-V4 region of the bacterial 16S rRNA genes was amplified using the primer pair: the forward S-D-Bact-0341-b-S-17 (CCTACGGGNGGCWGCAG) and the reverse S-D-Bact-0785-a-A-21 (GACTACHVGGGTATCTAATCC). The PCR cycling conditions were denaturation at 98 °C for 1 min, followed by 25 cycles at 98 °C for 10 s, 56 °C for 30 s, and 72 °C for 30 s, and a final extension at 72 °C for 2 min. The DNA amplicons were purified by solid-phase reversible immobilization on Agencourt AM Pure XP beads (Beckman Coulter, Brea, CA, USA), after which the quality of the DNA libraries was confirmed using the QIAxcel Advanced system (Qiagen GmbH, Hilden, Germany).

Prepared DNA libraries were sequenced on the Illumina MiSeq platform (Illumina Inc., San Diego, CA, USA), according to the manufacturer’s instructions. High-throughput paired-end sequencing was performed using the V.2 (300 bp × 2) reagent kit.

### 2.6. Bioinformatic Analysis

Raw paired-end sequencing reads were processed using USEARCH v11.0.667. Paired-end reads were merged using the -fastq_mergepairs command with a maximum of 10 mismatches (-fastq_maxdiffs 10) and a minimum percentage identity of 80% (-fastq_pctid 80). The merged reads were quality-filtered using the -fastq_filter command with a minimum sequence length of 420 bp (-minlen 420) and a maximum expected error rate of 1.0 (-maxee 1.0). The resulting sequences were dereplicated using the -fastx_uniques command.

Dereplicated sequences were clustered into operational taxonomic units (OTUs) using the -cluster_otus command at a 97% sequence-similarity threshold. Representative OTU sequences were subsequently matched against the processed sequence dataset using the -usearch_global command with the -strand plus option and a minimum sequence identity of 97% (-id 0.97).

After quality filtering, a total of 502,248 reads were retained from 24 cecal microbiomes. The number of retained reads per sample ranged from 11,131 to 31,809, with a mean of 20,927 ± 5079 reads per sample. No samples were excluded based on sequencing depth, as all samples contained more than 11,000 reads after quality filtering. Rarefaction curves reached a plateau, indicating adequate sequencing coverage for downstream analyses.

Taxonomic classification of the representative OTU sequences was performed using the RDP classifier [33] against the SILVA 16S rRNA database. Taxonomic assignments were reported at the phylum, class, order, family, and genus levels.

The resulting OTU abundance table and taxonomic annotations were imported into MicrobiomeAnalyst for downstream microbiome data processing, statistical analysis, and visualization.

To assess the amino-acid (AA)-related functional potential of the broiler chicken cecal microbiomes, PICRUSt2-based functional prediction was performed directly through the MicrobiomeAnalyst platform [34]. Low-frequency OTUs occurring at fewer than 4 reads in fewer than 20% of samples were removed prior to functional prediction. PICRUSt2 was used to infer microbial functional gene families and metabolic pathways from the 16S rRNA gene-based OTU profiles. Predicted functional profiles were characterized according to KEGG Orthology (KO) categories, and amino-acid metabolism-related KOs and pathways were subsequently analyzed.

Functional profiles were normalized using total-sum scaling. The results were expressed as the predicted relative representation of KEGG orthologs (KOs) associated with the metabolic functions of interest across the analyzed chicken groups.

Because PICRUSt2 infers microbial functional potential from 16S rRNA gene sequence data rather than directly measuring microbial genes, the predicted KOs and metabolic pathways were interpreted as estimates of functional potential and not as direct measurements of microbial gene abundance, gene expression, or metabolic activity. Accordingly, associations between predicted microbial functions and host amino-acid profiles were interpreted as potential associations rather than evidence of a causal role of the microbiota in regulating host amino-acid homeostasis.

### 2.7. Statistical Analysis

Experimental data, including the AA content in liver and thigh and breast muscles, as well as the microbial abundances in the gut microbiomes of each broiler chicken group, were pre-calculated as separate units and stored using Microsoft Excel 2019 package (Microsoft Corporation, Redmond, Washington, DC, USA). Advanced data processing was performed using Statistica 13.0 software (TIBCO Software Inc., Palo Alto, CA, USA).

Since the Shapiro–Wilk test rejected the normal distribution hypothesis of the original data at the 95% confidence level, nonparametric methods for subsequent statistical analysis were justified. In accordance with this approach, quantitative data were presented as median values [Q1–Q3], and differences in AA content between analyzed samples were assessed using the Wilcoxon matched pairs test. Downstream comparative analysis between the selected chicken groups was performed using the Mann–Whitney U-test, where the significance levels were considered to be 0.05, 0.01, and 0.001.

A multivariate statistical method called principal component analysis (PCA) was used to capture the largest variation in the AA content, and the distribution of chicken groups assigned to different AA profiles was visualized via an F1 × F2 factor plane.

Gut microbiome alpha diversity was estimated by the Chao, Shannon, and Simpson (1-D) indexes calculated using PAST V. 4.06 software. The distribution of alpha diversity indices, differences in the abundance of bacterial taxa between the selected chicken groups, and a comparison of the relative abundance of KOs in the gut microbiomes were assessed using the Wilcoxon matched pairs test. When comparing the relative abundance of bacterial taxa between selected chicken groups, a logarithmic transformation was performed.

Associations between AA profiles in various samples and bacterial taxa abundance in chicken-gut microbiomes were assessed by correlation analysis corrected for multiple comparisons and visualized via clustering “heat map” charts using the ChiPlot utility program (https://www.chiplot.online/) accessed on 1 June 2026. The resulting data on KEGG metabolic pathways were exported to Microsoft Excel for further statistical analysis and visualization using the MicrobiomeAnalyst 2.0 platform [34].

## 3. Results

### 3.1. Amino-Acid Composition of Liver and Meat Samples from Arbor Acres Broiler Chickens

Liver, thigh, and breast samples from 42-day-old Arbor Acres broiler chickens belonging to the 24 examined groups were analyzed for 14 individual amino-acid concentrations (Table 1).

The detailed AA composition of chicken liver and meat samples showed the highest concentrations of the branched-chain amino acids Leu + Ile, with absolute concentrations of these amino acids being statistically higher in breast compared to liver (*p* = 0.017720) and in thigh compared to liver (*p* = 0.000027), with no difference between thigh and breast. Additionally, the most abundant AAs generally found across all samples were flavor-related amino acids Ala and Arg, as well as the essential amino acid Lys, which was generally higher in muscle than in liver, while a statistically significant difference between thigh and breast was only observed for Ala (*p* = 0.045501). Moreover, breast meat had significantly higher aromatic amino-acid Tyr content compared to both thigh (*p* = 0.000967) and liver (*p* = 0.000021) samples. Against this background, no differences were found in the concentration of Phe in the compared substrates, and the null hypothesis regarding the distribution of Val and Pro between breast and liver samples was confirmed.

### 3.2. Amino-Acid Profiles of Chicken Liver and Meat Samples

**Figure 1:**
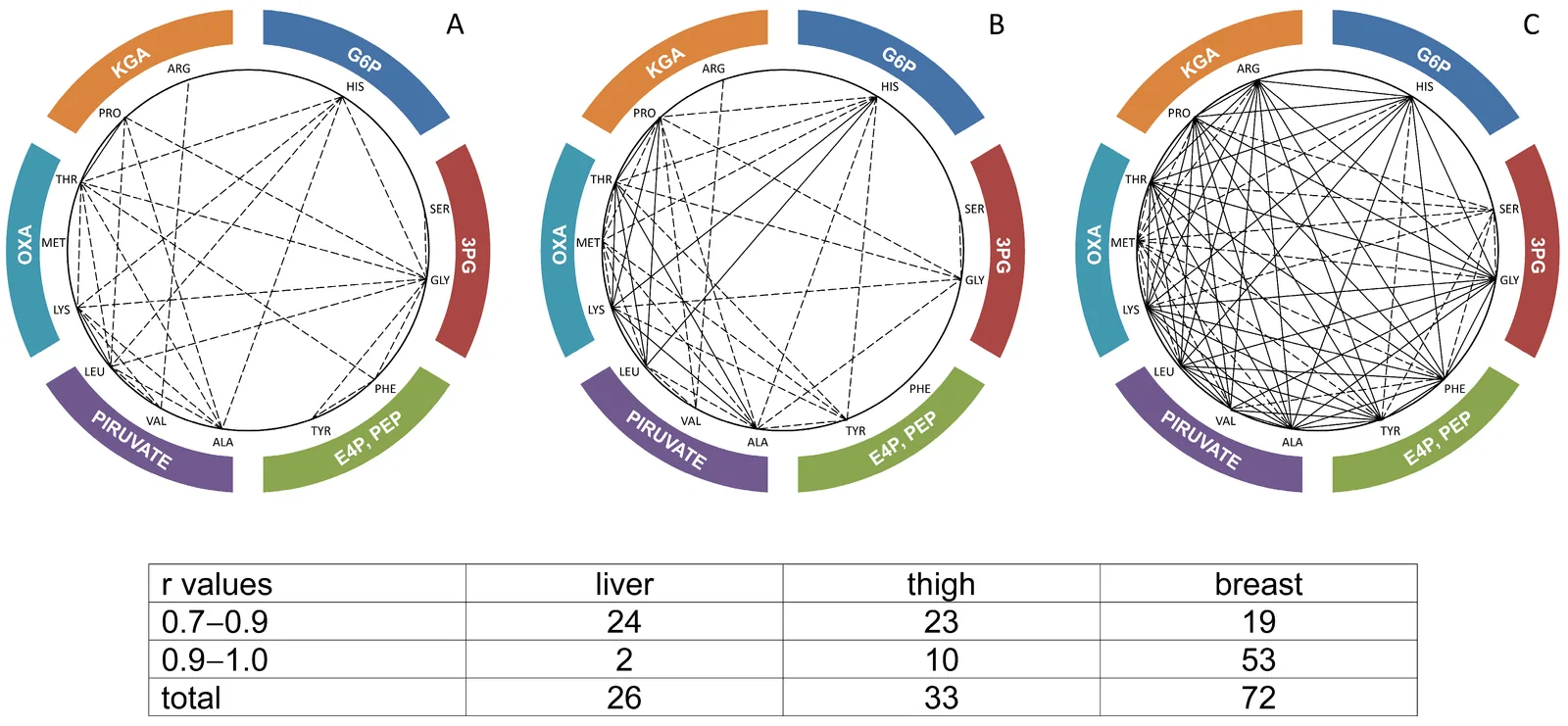
Amino acids co-abundance network in liver (**A**), thigh (**B**), and breast (**C**) samples collected from 24 Arbor Acres chicken groups. The correlation coefficients in the range of 0.7–0.9 are indicated by dotted lines, and the values in the range of 0.9–1.0 are indicated by solid lines.

Individual AA concentrations in broiler chickens demonstrated highly significant associations across different tissues (Figure 1). The evaluation of liver samples showed 26 positive values of the correlation coefficient (*p* < 0.001), revealing a co-abundance network in which 5 EAAs (His, Leu + Ile, Lys, Thr) were associated with each other and with other AAs by at least 6–7 links, and flavor-related amino acid Gly by 8 links, while Arg showed no such links. The examination of thigh samples showed the observed network as denser, with 33 significant correlation coefficients (including 10 extremely high ones in the range of 0.9–1.0). In turn, the breast samples demonstrated the highest integrity of AA composition, since the majority of calculated correlation coefficients were significant, including 19 links in the range of 0.7–0.9 and 53 in the range of 0.9–1.0.

Using principal component analysis (PCA), these data were transformed into a new coordinate system capturing the largest variation in the AA profiles across different tissues. For all substrates, the first principal component (PC1), which identifies the main trend of variability in the analyzed datasets, had significant unrotated factor loadings (>0.7) for most AAs included in the study (9 of 13 in liver, 10 of 13 in thigh, and 13 of 13 in breast). Accordingly, the correlation matrix eigenvalues and the PC1 total variances increased along the liver–thigh–breast line: from 7.580 to 11.825 and from 58.311% to 90.961%, respectively (Table 2).

The second principal component (PC2) calculations, which presented a linear combination of variables that captures the maximum remaining variance not explained by PC1, showed significant unrotated factor loadings for the flavor-related amino acid Arg in liver (0.778) and in thigh (0.754), whereas in breast all unrotated factor loadings were found to be insignificant (<0.7). Thus, PC2 in the liver (eigenvalue of the correlation matrix 2.735, total variance 21.038%) and in the thigh (eigenvalue of the correlation matrix 1.700, total variance 13.079%) were parameters that allowed us to separate the analyzed groups specifically in a direction perpendicular (orthogonal) to PC1 and independent of this tendency. On the other hand, for the breast samples, the PC2 eigenvalue of the correlation matrix was less than 1.0 (nominally 0.609), and the total variance (4.683%) was low, making it unsuitable for the chicken group discrimination.

**Figure 2:**
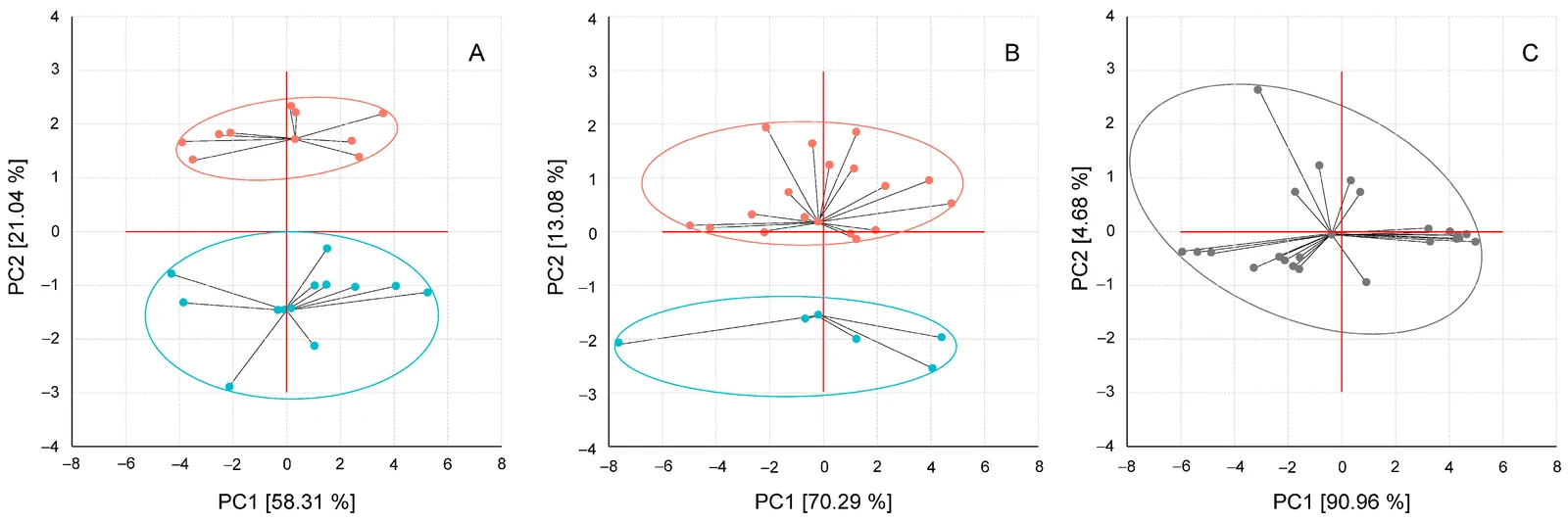
Projection of AA profiles in the liver (**A**), thigh (**B**), and breast (**C**) samples collected from 24 Arbor Acres chicken groups on the PC1 × PC2 factor planes, estimated using principal component analysis. In parts (**A**,**B**), Arg-high series are outlined with red lines, and Arg-low series are outlined with blue lines; in part (**C**), there is no separation into series; all groups are outlined with a gray line.

In accordance with the PCA algorithm, factor coordinates of cases (also known as scores or projections) were determined for each AA dataset, and these observations were subsequently classified based on positive or negative score values. The following projection of the observed scores onto the newly formed orthogonal PC1 × PC2 factor planes for the liver and thigh samples showed clear separation of the broiler chicken groups, while the breast samples were inseparable in this analysis (Figure 2C). Thus, based on the obtained factor coordinates and two-dimensional plot distribution, two series of liver and thigh samples were identified; each was linked with the PC2 factor directly or inversely. Since PC2 was significantly associated with the amino-acid Arg content (see above), these series were designated as Arg-high and Arg-low. According to this approach, 10 Arg-high groups and 14 Arg-low groups were identified between liver samples, while for thigh samples, the number of Arg-high data points increased to 18 and the number of Arg-low data points decreased to 6 (Figure 2A,B).

Comparison of Arg-high and Arg-low liver and thigh series revealed that the concentrations of several individual AAs differed significantly, exhibiting characteristic amino-acid profiles that exceeded the differences in Arg content alone (Table 3). Typically, the nonessential amino-acid Ser content decreased in the Arg-high series, which was shown for both liver (*p* = 0.001) and thigh (*p* = 0.045) samples. Moreover, Arg-high liver samples were enriched in Ala (*p* = 0.009), Lys (*p* = 0.037), and Leu + Ile (*p* = 0.046) but depleted in Tyr (*p* = 0.020) and Met (*p* = 0.002) content, whereas in the Arg-high thigh samples, decreased concentrations of the essential amino acid Phe were found (*p* = 0.018).

### 3.3. Cecal Microbiomes in Broiler Chickens with Different Liver and Meat Amino-Acid Profiles

Based on the analysis of a subset of 16S rRNA gene reads, 112 bacterial genera and equivalent taxonomic ranks, belonging to 36 families and 23 orders, combined into 16 classes and, at the highest taxonomic level, grouped into 8 phyla, were found in the chicken’s gut microbiomes.

**Figure 3:**
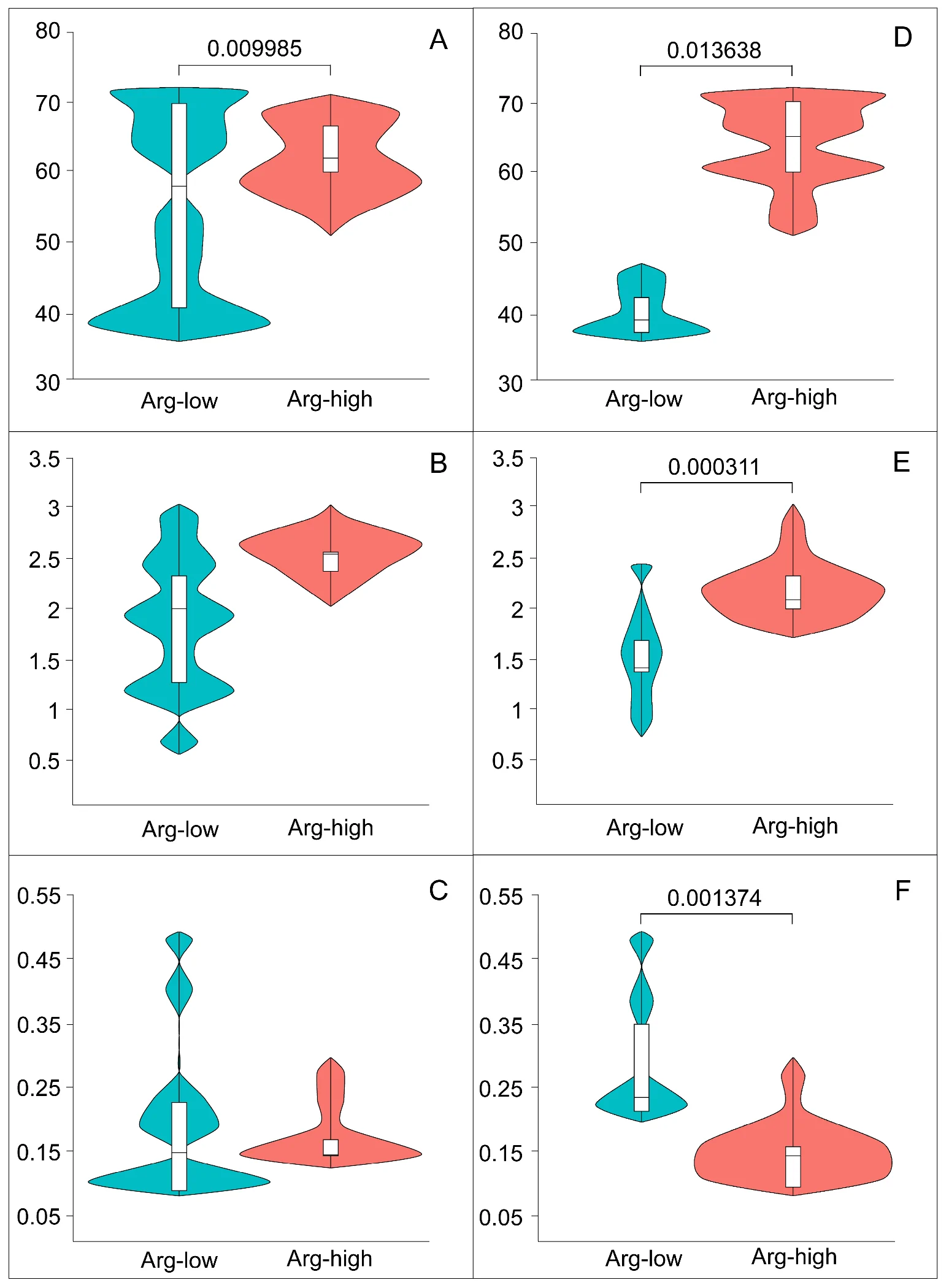
Comparison of alpha diversity indices (Chao1—(**A**,**D**); Shannon—(**B**,**E**); Simpson—(**C**,**F**)) between Arg-low and Arg-high liver (**A**–**C**) and thigh (**D**–**F**) series. Each panel superimposes box plots (M [Q1–Q3]) and density distributions. Datasets were analyzed using the Mann–Whitney U-test; statistically significant differences indicated.

When comparing the alpha diversity indices of the gut microbial community in Arbor Acres broiler chickens with different AA profiles, several trends were identified. As shown in Figure 3A,D, Chao1 index values, which estimate the total bacterial genera richness, were statistically significantly higher in the gut microbiomes belonging to Arg-high groups, both when comparing the liver series: 62.00 [60.00–66.50] vs. 58.00 [40.75–69.75]; *p* = 0.009985, and when comparing the thigh series: 65.00 [60.00–70.00] vs. 39.00 [37.25–42.25]; *p* = 0.013638. The Shannon index, which measures microbial alpha diversity taking into account both richness (number of genera) and evenness (relative abundance in the gut microbiome), revealed significant differences at *p* = 0.000311 between the Arg-low thigh groups (1.43 [1.39–1.70]) and the Arg-high thigh groups (2.10 [2.01–2.34]) whereas no significant differences were found between the Arg-low (2.01 [1.28–2.34]) and Arg-high (2.55 [2.38–2.57]) liver groups (Figure 3B,E). In turn, the Simpson index, which is more sensitive to evenness and dominant species number, showed no differences between the compared series of liver samples (Figure 3C), while it increased in the Arg-low thigh series vs. Arg-high thigh series: 0.23 [0.21–0.35] vs. 0.14 [0.09–0.16], respectively, at *p* = 0.001374 (Figure 3F).

To analyze the taxonomic composition and variations of the gut microbiota at the most detailed genus OTU level, a Mann–Whitney U-test was performed for the 20 most abundant gut microbial components in Arbor Acres broiler chickens.

As exhibited in Table 4, the relative abundance of the 10 observed genera, did not differ between the chicken’s microbiomes from the Arg-high and Arg-low liver series, while more unclassified *Oscillospiraceae* (*p* = 0.030) and *Lachnospiraceae* (*p* = 0.003), *Christensenella* (*p* = 0.030), *Eisenbergiella* (*p* = 0.004), *Dysosmobacter* (*p* = 0.007), *Anaerotignum* (*p* = 0.023), and *Oscillibacter* (*p* = 0.007), were observed across the Arg-high groups. In contrast, the chicken’s cecal microbiomes belonging to the Arg-low liver series were markedly enriched in the *Alistipes* genus abundance (*p* = 0.000021)—a feature not observed in the chicken’s microbiomes belonging to the Arg-high liver series—as well as by increased abundance of the *Lactobacillus* genus (*p* = 0.035). In turn, a comparison of Arg-high and Arg-low thigh series confirmed notable changes in the abundance of most observed genera and equivalent taxonomic ranks, showing differences at the highest statistical level (*p* < 0.001). Among differentially abundant taxa, the Arg-high thigh series showed a marked increase in 15 genera in addition to those described above, including *Butyricicoccus*, *Fournierella*, *Ligilactobacillus*, *Mediterraneibacter*, *Romboutsia*, *Subdoligranulum*, and *Turicibacter*.

**Figure 4:**
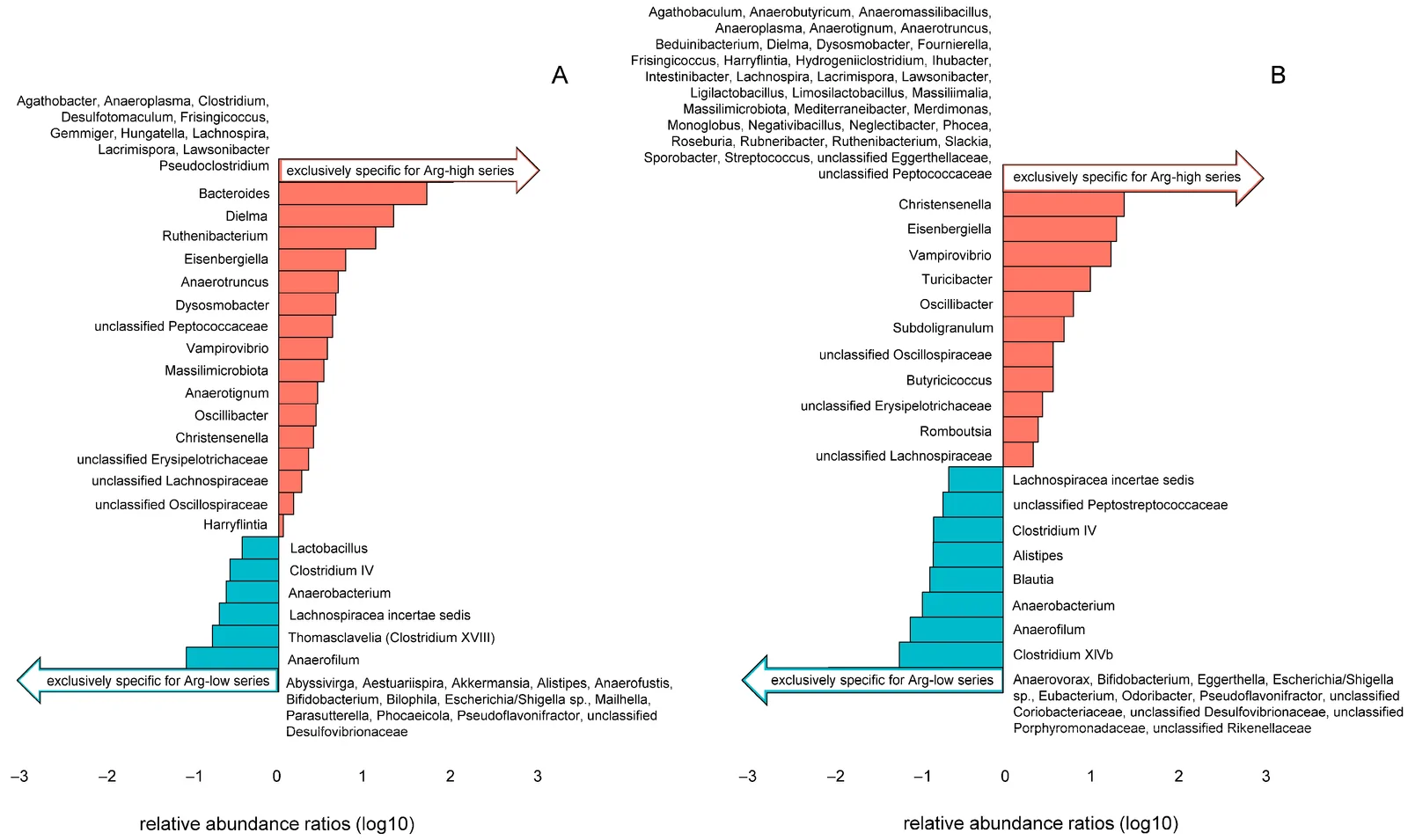
Relative abundance of bacterial taxa in the gut microbiomes belonging to Arg-low and Arg-high liver (**A**) and thigh (**B**) series. Bar plots represent log-transformed relative abundance ratios of bacterial taxa prevalent in Arg-high (red) and Arg-low (blue) series. Taxa that are exclusive to the corresponding series are indicated next to the arrows.

Represented by log-transformed relative abundance ratios, the gut microbiomes belonging to the Arg-high and Arg-low liver series showed significant differences across 46 genera and equivalent taxonomic ranks, including several minor taxa that were low in abundance and rare in only 1 to 10% of samples. Among them, 11 genera (*Agathobacter*, *Anaeroplasma*, *Clostridium*, *Desulfotomaculum*, *Frisingicoccus*, *Gemmiger*, *Hungatella*, *Lachnospira*, *Lacrimispora*, *Lawsonibacter* and *Pseudoclostridium*) were found exclusively in the gut microbiomes of broiler chickens with a high arginine content and were absent in the microbiomes of the Arg-low series. Another 16 taxa, including those presented in Table 4, showed statistically significant excess compared to the gut microbiomes of chickens belonging to the Arg-low series (Figure 4A). In turn, the gut microbiomes of chickens belonging to the Arg-low series showed the exclusively specific presence of 13 minor genera (*Abyssivirga*, *Aestuariispira*, *Akkermansia*, *Anaerofustis*, *Bifidobacterium*, *Bilophila*, *Escherichia/Shigella* sp., *Mailhella*, *Parasutterella*, *Phocaeicola*, *Pseudoflavonifractor* and unclassified *Desulfovibrionaceae*), as well as the exclusive dominance of the genus *Alistepes* (see above), and the relative abundance of 6 more taxa increased in the Arg-low series compared to the Arg-high series.

Comparison of gut microbiomes belonging to alternative chicken thigh series with different arginine contents increased the number of distinct taxa to 66, of which 36 were found exclusively in Arg-high groups and 11 in Arg-low groups (Figure 4B).

### 3.4. Heat Map Analysis of the Relationships Between Gut Microbiome Composition and Amino-Acid Profiles in Broiler Chicken Tissues

**Figure 5:**
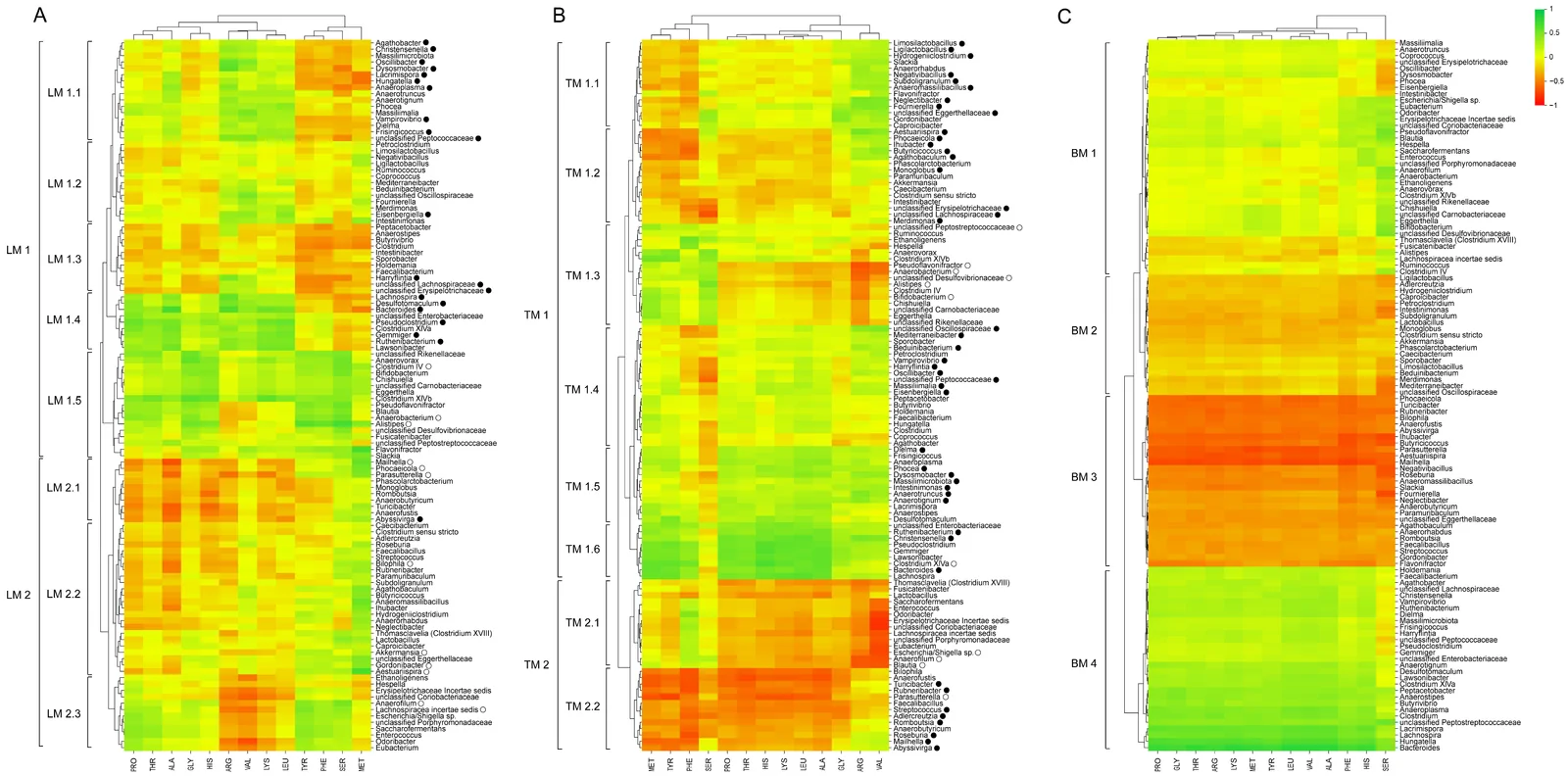
Clustered heat maps showing associations between the bacterial genera or equivalent taxonomic ranks abundance in chicken-gut microbiomes (lines) and amino-acid content (columns) in liver (**A**), thigh (**B**), and breast (**C**) samples collected from 24 Arbor Acres chicken groups. Bacterial taxa characteristic of the Arg-low series are indicated by white circles, and those of the Arg-high series are shown as black circles.

To provide a visual representation of the relationships between the AA profiles in the liver, thigh, and breast muscle tissues and the chicken-gut microbiome composition, three detailed heat maps of such distribution were constructed (Figure 5). These plots, combining Euclidean clustering with Z-score standardization, present the analyzed datasets based on individual amino-acid content (columns) and bacterial genera or equivalent taxa abundance (lines), color-coded highlighting positive (green) or negative (red) associations between these parameters.

In liver samples (Figure 5A), based on quantitative profiling of 14 amino acids, heat maps showed three distinct clusters following different bacterial abundance in the gut microbiomes. Among them, the central cluster combining two pairs of amino acids (Arg + Val/Lys + Leu) was essential for bacterial taxa clustering, which were divided into principal LM1 and LM2 clusters, mainly directly or inversely associated with the content of observed amino acids in liver tissue. In turn, each of these clusters was subdivided into five (LM1.1–LM1.5) and three (LM2.1–LM2.3) subclusters, respectively, which represented a mosaic nature of the relationships between the specific AA content and the bacterial taxa abundance.

All 33 genera and equivalent taxonomic ranks that showed significant abundance in the gut microbiomes of Arg-high liver series (see Figure 4A above) were found in cluster LM1 exclusively. These taxa were most frequently found in the LM1.1 subcluster, which was positively associated with the Arg + Val/Lys + Leu content and negatively with the Tyr + Phe/Ser/Met content, as well as in the LM1.4 subcluster, which was positively linked with both the Arg + Val/Lys + Leu and the Pro + Thr/Ala/Gly + His content. In the LM1.1 subcluster, there were 10 of 16 such taxa, including *Christensenella*, *Dysosmobacter*, *Oscillibacter* and *Vampirovibrio*, whereas in the LM1.4 subcluster there were 7 out of 9 taxa, including the genus *Bacteroides* and several minor genera. The genus *Eisenbergiella* was present in the LM1.2 subcluster; unclassified members of the *Erysipelotrichaceae* and *Lachnospiraceae* families, as well as the minor genus *Harryflintia,* were assigned to the LM1.3 subcluster; whereas no specific taxa of the gut microbiomes belonging to the Arg-high liver series were found in the LM1.5 subcluster.

In turn, most of the 13 bacterial taxa described as specific to the Arg-low liver groups were found in the LM2 cluster, namely: *Abyssivirga*, *Mailhella*, *Parasutterella* and *Phocaeicola* were assigned to the LM2.1 subcluster, which was negatively associated with both the Arg + Val/Lys + Leu and Pro + Thr/Ala/Gly + His content; *Aestuariispira*, *Akkermansia*, *Bilophila* and *Gordonibacter* belonged to LM2.2 subcluster; *Anaerofilum* and *Lachnospiracea* incertae sedis were present in LM2.3 subcluster. Additionally, *Alistipes*, *Anaerobacterium,* and *Clostridium IV* were found in the LM1.5 subcluster, positively associated with the highest amino-acid liver content, although just these genera did not show positive links with Arg + Val content.

When a heatmap linking the gut microbiome compositions and the breast AA profiles was generated (Figure 5B), the amino-acid clusters were rearranged, where the Arg + Val cluster occupied a peripheral position. In this mosaic heatmap, bacterial taxa specific to Arg-high and Arg-low thigh series were distributed more diffusely, while the first were frequently found in TM1.1, TM1.3, TM1.4, and TM2.2 subclusters and the second were present predominantly in TM1.2 and TM2.1 subclusters.

The heatmap constructed for the breast samples (Figure 5C) showed a non-mosaic pattern, as most amino acids (except Ser) were included in a single cluster, and bacterial taxa formed four robust clusters, uniquely associated with their content. In this distribution, total AA content was positively correlated with the abundance of 29 bacterial taxa (BM.4 cluster), which was dominated by the genus *Bacteroides*. In turn, for the BM.3 cluster, a strong negative correlation was found between the abundance of 27 bacterial genera in the chicken-gut microbiome and the AA content in breast muscles.

### 3.5. PICRUSt2-Based Prediction of Amino-Acid Metabolism-Related Functional Potential in Chicken Cecal Microbiomes

Among the 14 amino-acid metabolic pathways considered, eight pathways showed a detectable predicted functional representation in the chicken cecal microbiomes: (i) alanine, aspartate, and glutamate metabolism; (ii) glycine, serine, and threonine metabolism; (iii) cysteine and methionine metabolism; (iv) valine, leucine, and isoleucine biosynthesis; (v) lysine biosynthesis; (vi) arginine biosynthesis; (vii) histidine metabolism; and (viii) phenylalanine, tyrosine, and tryptophan biosynthesis. Amino-acid degradation pathways were not consistently represented in the predicted functional profiles or were detected only sporadically.

**Figure 6:**
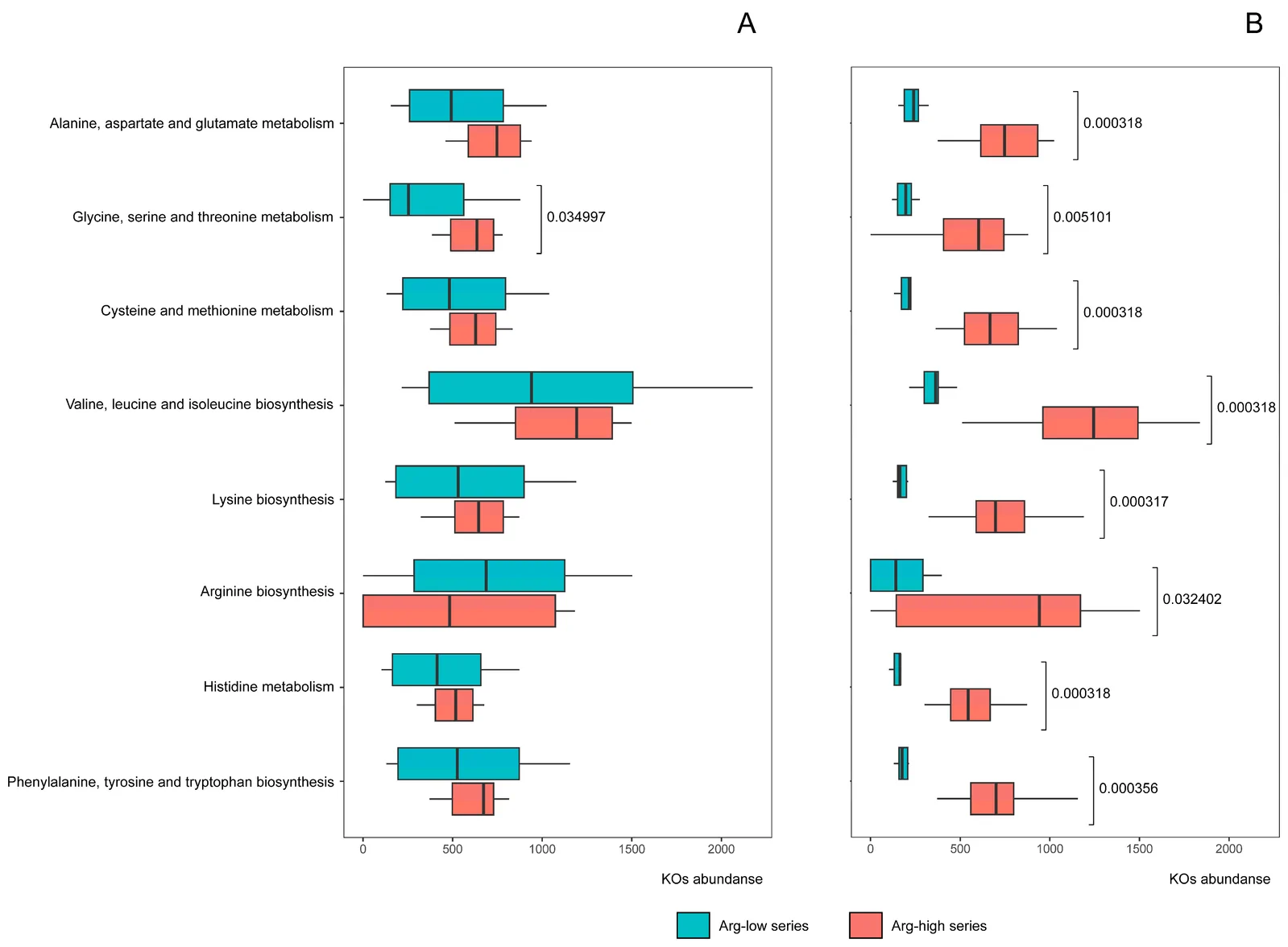
Comparison of the abundance of amino-acid metabolic pathways across Arg-low and Arg-high liver (**A**) and thigh (**B**) series. Datasets were presented as M [Q1–Q3] values and analyzed using the Mann–Whitney U-test; statistically significant differences indicated.

Comparison of the predicted relative representation of amino-acid metabolism-related KOs between the Arg-high and Arg-low liver series showed a tendency toward higher predicted functional representation in the Arg-high groups (Figure 6A). However, statistically significant differences were observed only for the glycine, serine, and threonine metabolism pathway, with values of 635.635 [461.120–758.010] and 254.410 [138.360–576.090] in the Arg-high and Arg-low groups, respectively (*p* = 0.034997). In turn, numerous statistically significant differences were found in the thigh series, regarding the predicted relative abundance of KOs associated with amino-acid biosynthetic pathways, which showed 3.3- to 4.9-fold higher predicted representation in Arg-high chickens than in Arg-low chickens (Figure 6B).

**Figure 7:**
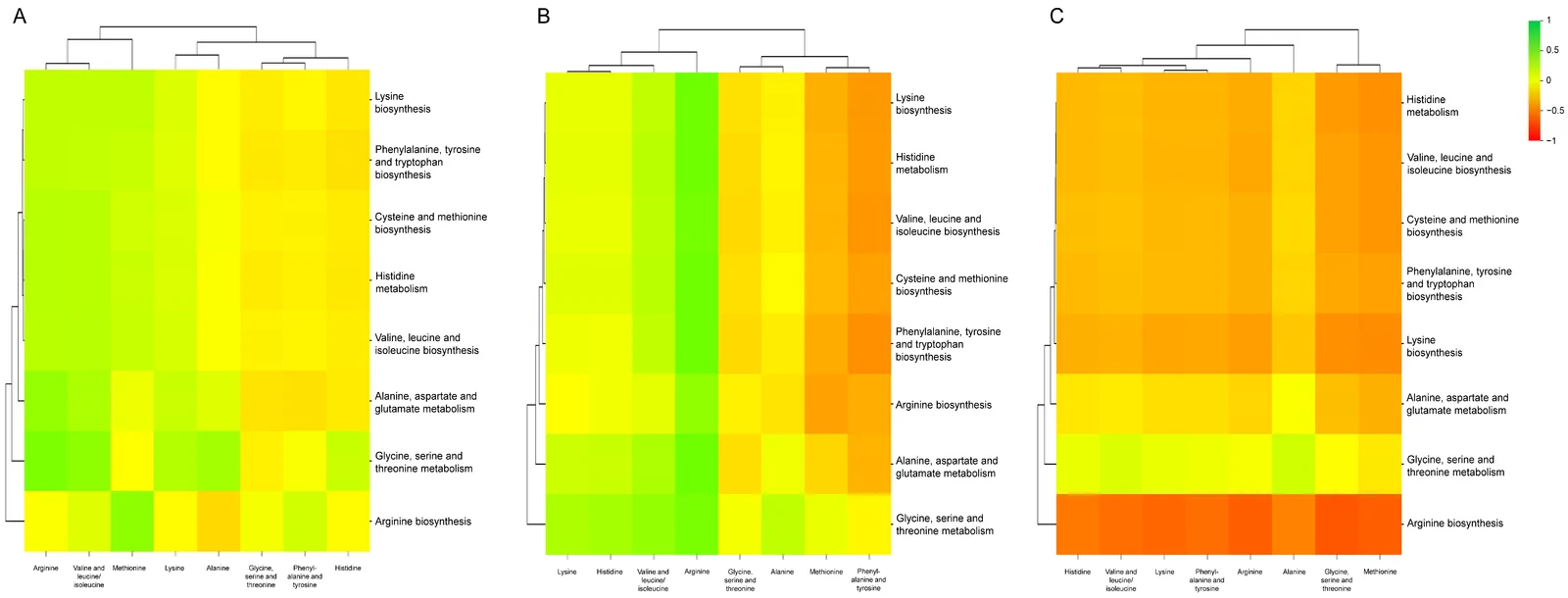
Heat maps showing the relationships between the relative abundance of amino-acid metabolic pathways in the gut microbiomes (lines) and amino-acid content (columns) in liver (**A**), thigh (**B**), and breast (**C**) samples collected from 24 Arbor Acres chicken groups.

To explore potential associations between the predicted microbial functional profiles and amino-acid concentrations measured in the liver, thigh, and breast muscles of Arbor Acres broiler chickens, heat maps were generated to visualize the relationships between selected KEGG pathways and the corresponding host amino-acid profiles (Figure 7).

An examination of liver/thigh amino-acid profiles and functional metabolic pathway profiles of the gut microbiomes showed a positive correlation between Arg and Val/Leu + Ile content (clustered together with Met in the liver heatmap or His and Lys in the thigh heatmap) and most KO relative abundance values. The other AA content in liver samples was weakly associated or independent of the potential functional capabilities of the gut microbiome (Figure 7A), whereas in thigh samples Met and Phe + Tyr content was negatively correlated with 7 of the 8 metabolic pathways (Figure 7B). In turn, breast AA profiles and microbiome metabolic profiles showed a predominantly inverse relationship, most notably for the Arg biosynthetic pathway, which was negatively associated with the content of all amino acids analyzed (Figure 7C).

## 4. Discussion

The initial stage of the current study focused on the examination of the amino-acid content of the Arbor Acres broiler chicken’s tissues belonging to different control and experimental groups fed with the use of various additives. Firstly, the resulting AA profiles showed the highest Leu content and increased concentrations of Ala, Arg and Lys, supporting the finding that Arbor Acres broiler products are a consistent source of a range of EAAs, BCAAs and FRAAs [35]. Second, individual AA contents (except Phe and, to some extent, Val and Pro) were typically higher in muscles than in liver samples. Differences were observed between thigh and breast muscles in the content of the flavor-related amino acid Ala and the aromatic amino acid Tyr. However, this variability was lower than that reported in D. D. Hamm’s 1981 study [36], a seminal piece of poultry science analyzing the nutritional and protein differences across different chicken loci.

Comparative analysis using principal component analysis revealed the existence of two distinct AA profiles in liver and thigh samples that differed significantly in Arg content, while all breast samples were similar in AA composition and could not be divided by the PCA algorithm. According to this approach, 10 Arg-high groups and 14 Arg-low groups were identified between liver samples, while for thigh samples, several data points were re-evaluated, and the ratio of Arg-high and Arg-low groups became 18/6, respectively. This novel finding seems significant because Arg is a strictly essential amino acid for broiler chickens’ rapid growth and muscle development, as they lack a complete urea cycle and cannot synthesize it de novo [37].

Since the broiler chicken diets of all control and experimental series had a stable AA content, and the feed additives used were targeted at the gut microbiomes, a likely explanation of this effect was a change in AA metabolism in the chicken’s gut [38], which spreads further along the microbiome–liver–muscle axis.

Metagenomic sequencing performed to test this hypothesis revealed the presence of 8 phyla, 16 classes, 23 orders, 36 families divided into 112 genera or equivalent taxonomic ranks, which is comparable with current data on the chicken-gut microbiome communities [39,40].

When comparing the taxonomic composition of the gut microbiomes of chickens with different AA profiles, the Arg-high series showed a predominance of bacterial genera belonging to the phylum *Bacillota* (formerly *Firmicutes* [41]), often described as components of the core microbiome [42] and beneficial for chicken performance. Thus, microbial communities in both Arg-high liver and thigh series were enriched with the *Eisenbergiella* genus, which provides better amino acid, nucleotide, and short-chain fatty acid metabolism [43] and is important for gut microbiota adaptation in the late stages of the broiler production cycle [44]. Similar data are available for unclassified *Lachnospiraceae* family members, which also ferment diverse plant polysaccharides to short-chain fatty acids (butyrate, acetate) and alcohols (ethanol) [45] and positively change economic indicators in poultry farming, e.g., feed conversion ratio [40]. The enrichment found in the genus *Oscillibacter* was seen as beneficial due to the fiber-fermenting activity to produce butyrate, which is critical for providing energy to the intestinal cells and maintaining gut barrier integrity [46]. An original observation was the detection of an unexpectedly high abundance of the genus *Christensenella*, known as beneficial gut bacteria for metabolic health in humans and associated with better weight management and greater disease resistance in chickens [47]. Together with numerous minor taxa, the gut microbial community of the Arg-high chicken groups showed increased alpha diversity Chao I and Shannon index values, typically indicating a more balanced and healthier microbiome, which strongly correlates with improved feed efficiency and highly efficient amino-acid processing in poultry [48].

In contrast, the gut microbiomes related to the Arg-low chicken groups were significantly enriched in the genus *Alistipes*, belonging to the phylum *Bacteroidota* (formerly *Bacteroidetes* [41]), the predominance of which resulted in a decrease in the Chao I and an increase in the Simpson index values. Interestingly, current research shows that *Alistipes* actively drives abdominal fat deposition and intramuscular fat accumulation in birds by altering bile acid metabolism and disrupting the amino acids tryptophan and histidine metabolism [49].

The relationship between individual microbiome members and tissue AA profiles was detailed in heat maps, demonstrating associations of each bacterial taxon with amino-acid content other than Arg only. For example, the above-mentioned taxa *Christensenella* and *Oscillibacter* were positively associated with the Arg + Val/Lys + Leu, while negatively associated with the Tyr + Phe/Ser/Met liver content, whereas unclassified members of the *Lachnospiraceae* family, as well as the genus *Bacteroides*, were positively linked with both the Arg + Val/Lys + Leu and the Pro + Thr/Ala/Gly + His content. In turn, the genus *Alistipes* was positively associated with the most (except Arg + Val) amino acids in liver content, while the most pronounced negative links between individual taxa and amino-acid profiles were found for numerous minor genera specific to microbiomes related to Arg-low chicken groups.

Notably, the heat maps constructed for the liver and thigh samples showed a mosaic format, demonstrating the unique metabolic effects of each bacterial taxon, while the heat map displaying the relationship between microbiome taxa and the amino-acid composition of breast muscles had a fundamentally different, non-mosaic format. Thus, although variations in the chicken-gut microbiome did not result in different AA profiles of chicken breast, the microbiome–host interaction in this system cannot be ignored and should be further investigated.

Since current research shows that extra-gut tissues and organs are profoundly impacted by gut-level host-microbe metabolic interactions [50], we reconstructed the AA metabolic pathways in the chicken-gut microbiomes. Based on the KEGG database resource [51], several pathways were identified, and their relative KO abundance was compared with observed AA content in chicken tissues. Notably, among the 14 metabolic pathways examined, microbial catabolism and amino-acid utilization were absent or sporadically detected, while biosynthetic pathways were consistently detected, suggesting de novo synthesized microbiome-derived AAs as a possible source of building blocks for host proteins [52]. In support, it has been determined that the most enriched Val and Leu + Ile biosynthesis pathway, as well as the Arg biosynthesis pathway in chicken-gut microbiomes, corresponded well with the highest concentrations of the BCAAs leucine/isoleucine and FRAA arginine experimentally detected in the chicken liver and meat samples. Previously, a significant positive correlation between the abundance of microbial genes involved in BCAA biosynthesis and host plasma levels was described by Hayashi et al. [53], and a vital role of the gut microbiome in Arg biosynthesis has been reported in Kang et al.’s study [54], indicating a universal gut–muscle axis in animals and humans for maintaining muscle weight and muscle fiber integrity.

Comparison of microbiome metabolic profiles of Arg-high series vs. Arg-low groups revealed a trend toward significant enrichment of biosynthetic pathways, reflecting the overall AA requirement for muscle protein accretion in growing chickens with arginine availability [55]. Following analysis of microbiome metabolic profiles and the AA profiles in chicken tissues also showed a number of positive links with Arg and Val/Leu + Ile content in liver and thigh samples, although these associations were semi-specific and involved most amino-acid biosynthetic pathways. At the same time, a number of microbiome–host links were not confirmed or showed inverse trends, especially in the analysis of breast samples, where AA content was inversely associated with the abundance of AA metabolic pathways in the chicken gut, as previously described in several multi-omics chicken studies [56,57].

In discussing the methodological limitations of this study, it should be noted that the direct impact of the dietary supplements on the host was not taken into account; instead, the diet was considered solely as an external factor influencing the microbiome. In this regard, the described effects can be viewed as part of an integrative associative analysis within the diet–microbiome–host system [58] that requires further refinement through a comparison of this system with a two-component diet–host system using germ-free birds.

Another methodological limitation is that the microbiome’s functional potential was predicted solely based on 16S rRNA sequencing data, rather than through direct measurements of actual metabolic genes, transcripts, proteins, or metabolites [59]. The authors hope that subsequent multi-omics studies will allow for a more detailed characterization of the microbiome–host interrelationships, and they intend to contribute to this effort in future research.

At the same time, these aspects do not negate the overall study results, which include the description of typical AA profiles in chicken liver and muscle tissues, as well as their associations with the gut microbiome composition, but they may significantly clarify the mechanisms involved, making the microbiome–host dependencies more understandable and predictive.

## 5. Conclusions

In fast-growing broiler chickens of the Arbor Acres strain, two distinct amino-acid profiles have been described for the liver and thigh muscle, which differ in Arg content and vary in some other AAs, while these differences for the breast muscle were not found. The gut microbiomes of chickens with Arg-high profiles showed an increased Chao I and Shannon diversity index, which was determined by a significant abundance of various members of the phylum *Bacillota*. In turn, the gut microbial communities of chickens with Arg-low profiles tended to have a decreased Chao I index and increased Shannon dominance index values, which was explained by the significant abundance of the genus *Alistipes* belonging to the phylum *Bacteroidota*. These microbiome differences resulted in enrichment or depletion of several AA biosynthetic pathways in the chicken gut, which appears to be an important reason for the distinct microbiome-related AA profiles in the liver and muscle tissue of Arbor Acres broiler chickens.

## Figures and Tables

**Table 1:** Individual amino-acid content in liver, thigh, and breast samples collected from 24 Arbor Acres chicken groups.

Amino Acids	Amino Acids Content M [Q1–Q3], mg/g			Wilcoxon Matched Pairs Test *p*-Level		
	Liver (L)	Thigh (T)	Breast (B)	L-T	L-B	T-B
Ribose-5-phosphates:						
**HIS**	15.75 [14.50–16.75]	22.00 [20.90–23.60]	25.35 [18.95–29.50]	**0.000018**	**0.000030**	>0.05
3-Phosphoglycerates:						
**SER**	23.00 [20.00–25.75]	27.10 [25.20–30.75]	27.50 [22.65–32.80]	**0.000091**	**0.006194**	>0.05
**GLY**	31.00 [29.50–33.00]	37.90 [35.45–41.45]	38.90 [28.80–42.00]	**0.000027**	**0.011928**	>0.05
Erythrose 4-phosphate and phosphoenolpyruvates:						
**PHE**	30.00 [27.50–31.50]	30.50 [27.85–33.85]	34.00 [24.15–36.50]	>0.05	>0.05	>0.05
**TYR**	24.00 [22.00–25.60]	31.50 [27.55–35.00]	40.00 [30.35–49.50]	**0.000034**	**0.000021**	**0.000967**
Pyruvates:						
**ALA**	40.50 [37.70–44.70]	56.55 [50.55–61.20]	65.40 [47.90–77.00]	**0.000018**	**0.000018**	**0.045501**
**VAL**	32.05 [30.00–35.60]	40.15 [35.90–43.15]	36.50 [22.35–43.00]	**0.000127**	>0.05	>0.05
**LEU + ILE**	79.95 [74.40–85.00]	99.50 [92.55–108.50]	98.35 [64.80–116.90]	**0.000027**	**0.017720**	>0.05
Oxaloacetate/aspartates:						
**LYS**	40.00 [38.00–46.60]	71.50 [67.50–76.55]	75.00 [53.65–84.75]	**0.000018**	**0.000018**	>0.05
**MET**	17.95 [15.45–20.45]	20.90 [18.40–26.00]	23.20 [16.15–26.50]	**0.003906**	**0.023122**	>0.05
**THR**	28.80 [27.50–30.90]	37.70 [35.50–41.00]	38.80 [27.75–43.45]	**0.000018**	**0.001136**	>0.05
α-Ketoglutarates:						
**PRO**	28.00 [26.80–29.50]	31.95 [29.50–35.50]	30.05 [23.45–34.00]	**0.000255**	>0.05	>0.05
**ARG**	40.85 [36.75–45.50]	55.45 [50.85–59.50]	54.30 [40.15–59.50]	**0.000018**	**0.000112**	>0.05

**Table 2:** Principal component analysis data assessed the largest variation in the AA profiles across liver, thigh, and breast samples collected from 24 Arbor Acres chicken groups.

Unrotated Factor Loadings for Individual AAs:	Liver		Thigh		Breast	
	PC1	PC2	PC1	PC2	PC1	PC2
**HIS**	**0.893**	−0.079	**0.922**	−0.028	**0.944**	−0.125
**SER**	0.521	−0.694	0.662	−0.296	**0.782**	0.601
**GLY**	**0.929**	−0.231	**0.859**	0.034	**0.986**	−0.036
**PHE**	**0.840**	−0.425	0.639	−0.562	**0.977**	0.076
**TYR**	**0.708**	−0.603	**0.816**	−0.395	**0.940**	−0.029
**ALA**	**0.784**	0.427	**0.956**	−0.050	**0.969**	−0.196
**VAL**	0.660	0.448	**0.710**	0.592	**0.960**	−0.104
**LEU + ILE**	**0.892**	0.362	**0.930**	0.200	**0.989**	−0.105
**LYS**	**0.848**	0.417	**0.972**	0.040	**0.989**	−0.051
**MET**	0.421	−0.590	**0.810**	−0.384	**0.883**	0.369
**THR**	**0.931**	0.001	**0.981**	−0.004	**0.994**	−0.045
**PRO**	**0.830**	0.090	**0.928**	0.171	**0.990**	−0.075
**ARG**	0.381	**0.778**	0.573	**0.754**	**0.974**	−0.130
**Eigenvalue of the correlation matrix**	7.580	2.735	9.137	1.700	11.825	0.609
**Total explained variance (%)**	58.311	21.038	70.288	13.079	90.961	4.683

**Table 3:** Comparison of AA profiles in liver and thigh samples belonging to Arg-low and Arg-high Arbor Acres chicken series.

Amino Acids	Liver			Thigh		
	ARG-Low Groups M [Q1–Q3]	ARG-High Groups M [Q1–Q3]	Mann–Whitney U-Test *p*-Level	ARG-Low Groups M [Q1–Q3]	ARG-High Groups M [Q1–Q3]	Mann–Whitney U-Test *p*-Level
Ribose-5-phosphates:						
**HIS, mg/g**	15.85 [14.00–16.88]	15.50 [15.00–16.00]	>0.05	21.40 [20.58–22.38]	22.00 [21.10–23.90]	>0.05
3-Phosphoglycerates:						
**SER, mg/g**	25.35 [23.25–28.53]	20.00 [19.00–22.00]	**0.001**	31.70 [27.88–32.68]	27.00 [24.33–27.85]	**0.045**
**GLY, mg/g**	31.00 [30.00–33.45]	30.50 [29.25–32.00]	>0.05	36.40 [34.25–40.43]	38.00 [36.25–41.45]	>0.05
Erythrose 4-phosphate and phosphoenolpyruvates:						
**PHE, mg/g**	30.00 [29.03–32.38]	28.00 [27.00–31.00]	>0.05	34.15 [31.83–37.60]	30.00 [27.48–31.78]	**0.018**
**TYR, mg/g**	25.25 [23.18–27.10]	22.50 [20.50–24.00]	**0.020**	33.50 [27.80–37.85]	30.85 [28.15–33.75]	>0.05
Pyruvates:						
**ALA, mg/g**	39.50 [35.63–41.95]	43.50 [41.25–46.50]	**0.009**	50.35 [47.73–58.30]	57.55 [53.38–61.50]	>0.05
**VAL, mg/g**	32.00 [28.90–33.00]	34.50 [31.25–36.75]	>0.05	35.40 [25.40–39.33]	41.50 [38.85–43.23]	>0.05
**LEU + ILE, mg/g**	77.45 [73.20–80.00]	81.50 [77.75–88.00]	**0.046**	92.85 [81.50–96.55]	102.00 [95.55–108.75]	>0.05
Oxaloacetate/aspartates:						
**LYS, mg/g**	39.05 [37.33–40.00]	44.00 [42.25–48.00]	**0.037**	70.00 [63.73–72.98]	71.95 [69.08–76.78]	>0.05
**MET, mg/g**	19.80 [18.05–21.75]	15.50 [14.25–17.00]	**0.002**	23.40 [20.58–28.25]	20.90 [18.25–25.50]	>0.05
**THR, mg/g**	28.55 [28.00–30.43]	29.00 [27.25–31.75]	>0.05	36.90 [33.75–40.20]	38.30 [36.00–40.75]	>0.05
α-Ketoglutarates:						
**PRO, mg/g**	27.00 [26.75–28.90]	29.00 [27.25–30.75]	>0.05	30.55 [28.03–33.98]	32.40 [30.00–35.75]	>0.05
**ARG, mg/g**	38.05 [35.38–39.88]	46.50 [44.25–48.00]	**0.001**	44.85 [43.85–50.58]	56.85 [54.25–60.75]	**0.006**

**Table 4:** The 20 most abundant bacterial genera and equivalent taxonomic ranks found in the gut microbiomes of Arbor Acres broiler chickens: comparison between Arg-low and Arg-high series.

Genera or Equivalent Taxonomic Ranks	Liver			Thigh		
	ARG-Low Groups	ARG-High Groups	Mann–Whitney U-Test *p*-Level	ARG-Low Groups	ARG-High Groups	Mann–Whitney U-Test *p*-Level
unclassified *Oscillospiraceae*	3181.00 [1267.00–4275.00]	4686.75 [4354.00–4760.00]	**0.030**	1246.75 [927.00–1510.00]	4655.75 [3982.00–4760.00]	**<0.001**
*Alistipes*	2932.00 [1379.00–5970.50]	0.00 [0.00–0.00]	**<0.001**	7063.25 [4357.00–10324.00]	0.00 [0.00–1629.00]	**<0.001**
*Bacteroides*	109.50 [49.00–826.50]	5461.50 [4372.00–8241.00]	**<0.001**	849.25 [542.00–884.00]	3092.75 [56.00–5483.50]	>0.05
*Faecalibacterium*	752.50 [459.00–1175.00]	1004.00 [579.00–2691.50]	>0.05	887.50 [487.50–1175.00]	839.25 [502.00–1613.00]	>0.05
unclassified *Lachnospiraceae*	572.75 [352.00–760.00]	1050.50 [737.00–1391.00]	**0.003**	414.50 [271.50–538.00]	916.75 [697.00–1355.00]	**0.002**
*Christensenella*	424.00 [45.00–695.00]	1063.75 [387.00–2032.00]	**0.030**	29.75 [12.00–45.50]	722.50 [418.00–1197.00]	**<0.001**
*Lactobacillus*	611.00 [306.00–1035.00]	233.25 [132.50–460.00]	**0.035**	611.00 [364.00–821.00]	288.75 [132.50–622.00]	>0.05
*Eisenbergiella*	125.00 [46.00–332.00]	733.75 [589.00–1023.00]	**0.004**	32.00 [18.00–51.50]	634.75 [307.00–1023.00]	**<0.001**
*Mediterraneibacter*	121.00 [0.00–423.00]	414.00 [291.00–675.00]	>0.05	0.00 [0.00–0.00]	409.00 [273.00–675.00]	**<0.001**
*Dysosmobacter*	71.50 [0.00–280.00]	323.50 [241.50–424.00]	**0.007**	0.00 [0.00–0.00]	279.00 [180.00–379.00]	**<0.001**
*Intestinimonas*	228.00 [0.00–381.00]	150.75 [0.00–556.50]	>0.05	2.00 [0.00–9.50]	314.50 [0.00–529.00]	>0.05
*Butyricicoccus*	239.50 [77.00–508.00]	218.75 [155.00–307.00]	>0.05	75.25 [34.00–138.00]	281.00 [170.00–460.00]	**0.002**
*Subdoligranulum*	397.50 [81.50–437.00]	98.00 [83.00–254.00]	>0.05	70.25 [37.00–98.00]	351.25 [91.00–437.00]	**0.009**
*Ruminococcus*	180.25 [67.00–306.00]	150.75 [66.00–222.00]	>0.05	248.00 [170.50–314.00]	116.75 [59.00–239.00]	>0.05
*Anaerotignum*	111.00 [0.00–199.00]	308.50 [120.00–451.00]	**0.023**	0.00 [0.00–0.00]	224.50 [120.00–375.50]	**<0.001**
*Ligilactobacillus*	20.50 [0.00–236.00]	226.25 [102.00–276.50]	>0.05	0.00 [0.00–0.00]	224.75 [51.50–521.00]	**<0.001**
*Fournierella*	47.00 [0.00–210.00]	128.00 [72.00–202.50]	>0.05	0.00 [0.00–0.00]	199.00 [72.00–212.00]	**<0.001**
*Turicibacter*	91.25 [16.00–1002.00]	102.00 [40.00–236.00]	>0.05	16.00 [3.50–30.00]	159.00 [93.00–994.00]	**0.002**
*Romboutsia*	87.00 [67.00–309.00]	109.00 [89.00–213.00]	>0.05	51.75 [24.50–73.00]	130.00 [97.00–309.00]	**0.005**
*Oscillibacter*	48.25 [21.00–96.00]	129.25 [114.00–145.00]	**0.007**	18.50 [3.00–29.50]	117.50 [80.00–137.50]	**<0.001**

## Data Availability

The data presented in this study are available on request by e-mail to icis-ofrc@list.ru, belonging to the Institute for Cellular and Intracellular Symbiosis (ICIS) of the Ural Branch of the Russian Academy of Sciences (Orenburg, Russia).

## Abbreviations

The following abbreviations are used in this manuscript:

AAs	Amino acids
EAAs	Essential amino acids
BCAAs	Branched-chain amino acids
NEAAs	Nonessential amino acids
FRAAs	Flavor-related amino acids
BD	Basal diet
PTC	Phenylthiocarbamyl
Arg	Arginine
Lys	Lysine
Tyr	Tyrosine
Phe	Phenylalanine
His	Histidine
Met	Methionine
Val	Valine
Pro	Proline
Ala	Alanine
Gly	Glycine
Leu + Ile	Leucine and Isoleucine
PCA	Principal component analysis
OTUs	Operational taxonomic units
GTDB	Genome taxonomy database
KEGG	Kyoto Encyclopedia of Genes and Genomes
KOs	KEGG orthologs
